# PCH'ing Together an Understanding of Crossover Control

**DOI:** 10.1371/journal.pgen.1000576

**Published:** 2009-07-24

**Authors:** Drew Thacker, Scott Keeney

**Affiliations:** 1 Molecular Biology Program, Memorial Sloan-Kettering Cancer Center, New York, New York, United States of America; 2 Weill Graduate School of Medical Sciences, Cornell University, New York, New York, United States of America; 3 Howard Hughes Medical Institute, New York, New York, United States of America; The University of North Carolina at Chapel Hill, United States of America

Meiosis is a specialized form of cell division involving one round of chromosome replication followed by two rounds of segregation, thereby producing daughter cells with half the genomic equivalent of the progenitor. In most organisms, double-strand breaks (DSBs) are introduced into the genome following premeiotic S-phase. These breaks are repaired almost exclusively from the homologous chromosome via repair pathways that yield either a crossover or non-crossover recombination product [1],[2]. Of particular importance are the crossovers, which tether homologous chromosomes and ensure accurate segregation at the first meiotic division (MI) [3]. Chromosomes that fail to cross over have significantly higher rates of non-disjunction at MI, which produces aneuploid gametes, causing miscarriages and birth defects in humans.

It should be no surprise then that most eukaryotes possess a sophisticated mechanism to control meiotic recombination. Consider the situation in an individual mouse meiocyte. More than 200 DSBs are made; however, only a subset of these precursors are repaired as crossovers, while the rest are repaired as non-crossovers (Figure 1) [4]. Thus, central to the “crossover control” mechanism is a decision to direct a given DSB to either a crossover or non-crossover fate [5]. This process ensures that each homolog pair receives at least one crossover (often referred to as the obligate crossover), and also regulates the spatial distribution of crossovers along chromosomes such that, if a chromosome receives two or more crossovers, they tend to occur further apart than expected by chance (referred to as crossover interference) (Figure 1B) [6]. Furthermore, it has been shown that when the number of DSBs is reduced, crossovers tend to be maintained at the expense of non-crossovers (a phenomenon called crossover homeostasis) [7],[8]. It has been proposed that the obligate crossover, crossover interference, and crossover homeostasis are all manifestations of a single or closely related set of molecular processes, but this hypothesis remains to be rigorously tested [7],[9],[10].

**Figure 1:**
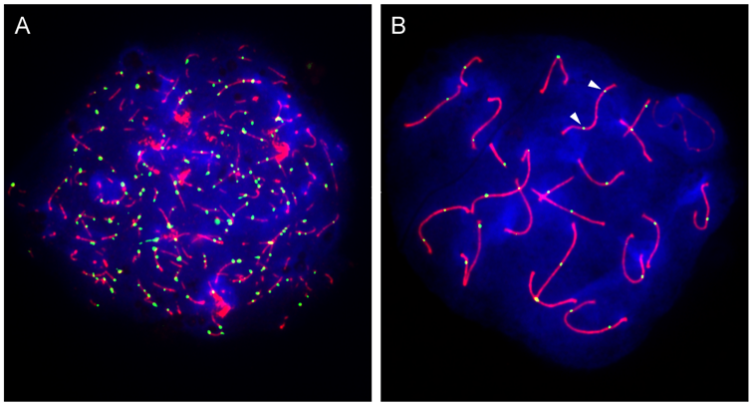
Only a subset of DSBs become crossovers. Mouse spermatocyte spreads were stained for the chromosomal axial element SYCP3 (red) and either (A) RAD51 (green) or (B) MLH1 (green). DAPI staining is shown in blue. Each DSB gives rise to a chromosome-associated RAD51 complex, whereas MLH1 complexes localize only to sites that will become crossovers. There is an approximate 9-fold excess of DSB-associated foci relative to crossover-associated foci. Arrowheads point to an example of an autosome with two widely separated MLH1 foci, characteristic of crossover interference. (Images courtesy of Ignasi Roig, Molecular Biology Program, Memorial Sloan-Kettering Cancer Center).

Almost a century after the first observation of crossover control [11], we still know very little about the underlying mechanism(s). Most of the proteins that have been shown to influence crossover control in budding yeast appear to function downstream of the crossover/non-crossover decision. One such class of proteins, commonly referred to as ZMMs (Zip1/2/3/4, Msh4/5, Mer3), is specifically required for the repair of DSBs into crossovers that exhibit interference [2]. Deletion of any of the ZMM genes causes accumulation of intermediates in the crossover pathway and subsequent prophase arrest [12].

Two articles in this issue of *PLoS Genetics* identify a role for the Pachytene Checkpoint gene, *PCH2*, in crossover control in *Saccharomyces cerevisiae* [13],[14]. *PCH2*, which encodes a putative AAA+-ATPase, was initially identified in yeast as a checkpoint factor due to suppression of a *zip1Δ* arrest in a *pch2Δ* mutant. This and other observations led to the hypothesis that Pch2 helps monitor chromosome synapsis during meiotic prophase [15],[16]. However, studies in yeast, flies, and mice revealed that Pch2 is not just a checkpoint factor, but that it is also required for chromosome axis organization and DSB repair [17],[18],[19]. Interestingly, *PCH2* is widely conserved in organisms that construct a synaptonemal complex and exhibit crossover interference, but is absent from organisms such as *Schizosaccharomyces pombe* that do not exhibit these features [15]. This observation suggested that Pch2 might also function in crossover control. Recent analysis in yeast demonstrated a small reduction in crossover numbers in *pch2* mutants at the *HIS4LEU2* recombination hotspot [19], but data available at the time did not allow evaluation of crossing over genome-wide and also did not address whether crossover control was normal.

In studies published in this issue of *PLoS Genetics*, the Alani and Börner groups [13],[14] have examined these issues in detail. When crossover frequencies were measured across several genetic intervals on chromosomes III, VII, and VIII, Zanders et al. [13] and Joshi et al. [14] observed either no difference or an increase (depending on the interval) in *pch2Δ* strains. Importantly, these analyses demonstrated that the crossovers formed in *pch2Δ* mutants show reduced interference.

Recent studies have indicated that decreased crossover interference is associated with a concomitant decrease in crossover homeostasis [8]. To investigate this relationship, both Zanders et al. and Joshi et al. measured spore viability of *pch2Δ* strains carrying various hypomorphic alleles of the topoisomerase-like protein, Spo11. These hypomorphic alleles decrease the number of DSBs [20]. If crossover homeostasis and crossover interference are separate manifestations of a common crossover control mechanism, then an interference-defective mutant would also be expected to show defects in homeostasis, and thus a decrease in DSBs in such a mutant should result in fewer crossovers that are randomly distributed throughout the genome. Such a scenario would in turn be expected to result in an increase in the frequency of chromosome pairs without a crossover, causing reduced spore viability because of MI non-disjunction. Indeed, although *pch2* mutation has little or no effect on spore viability on its own, introducing a *spo11* mutation that reduces DSB activity by ∼20% significantly reduced viability despite approximately wild-type crossover frequencies [13],[14]. This reduction in spore viability was further exacerbated in *spo11* hypomorphs that reduce DSB activity up to 80%. Although this is an indirect method of measuring crossover homeostasis, these findings provide compelling evidence that Pch2 has a role in multiple aspects of crossover control during yeast meiosis.

So what role could Pch2 play in this process? Pch2 is required for differential organization of chromosome structural proteins Hop1 and Red1 relative to the synaptonemal complex central element protein Zip1 [19]. In *pch2Δ* mutants, Hop1/Red1 and Zip1 exhibit a more uniform axial localization pattern than is observed in wild type. Joshi et al. now demonstrate that chromosome domains that are enriched for Hop1 and Red1 tend to colocalize with future sites of crossover formation, leading to the hypothesis that Pch2 functions to stabilize alternating domains enriched for either Hop1/Red1 or Zip1. Such domains are proposed to be modules that mediate crossover designation and interference. Interestingly, when *PCH2* is deleted, not only is axial organization of Hop1/Red1 and Zip1 compromised, but appearance of both crossover and non-crossover products is delayed to similar extents [19]. It is not yet clear whether these different aspects of the *pch2* mutant phenotype are consequences of the same molecular defect, nor is it yet clear precisely how Pch2 protein functions in wild-type cells. Nonetheless, the current findings provide new support for the idea that higher order chromosome structure plays a key role in crossover control [9], and furthermore implicate Pch2 as an important player in coordinating recombination with large-scale chromosome structures.

## References

[1] Allers T, Lichten M (2001). Differential timing and control of noncrossover and crossover recombination during meiosis.. Cell.

[2] Borner GV, Kleckner N, Hunter N (2004). Crossover/noncrossover differentiation, synaptonemal complex formation, and regulatory surveillance at the leptotene/zygotene transition of meiosis.. Cell.

[3] Page SL, Hawley RS (2003). Chromosome choreography: The meiotic ballet.. Science.

[4] Buhler C, Borde V, Lichten M (2007). Mapping meiotic single-strand DNA reveals a new landscape of DNA double-strand breaks in *Saccharomyces cerevisiae*.. PLoS Biol.

[5] Bishop DK, Zickler D (2004). Early decision; Meiotic crossover interference prior to stable strand exchange and synapsis.. Cell.

[6] Jones GH (1984). The control of chiasma distribution.. Symp Soc Exp Biol.

[7] Martini E, Diaz RL, Hunter N, Keeney S (2006). Crossover homeostasis in yeast meiosis.. Cell.

[8] Chen SY, Tsubouchi T, Rockmill B, Sandler JS, Richards DR (2008). Global analysis of the meiotic crossover landscape.. Dev Cell.

[9] Kleckner N, Zickler D, Jones GH, Dekker J, Padmore R (2004). A mechanical basis for chromosome function.. Proc Natl Acad Sci U S A.

[10] Hillers KJ (2004). Crossover interference.. Curr Biol.

[11] Muller HJ (1916). The mechanism of crossing over.. Am Nat.

[12] Lynn A, Soucek R, Borner GV (2007). ZMM proteins during meiosis: Crossover artists at work.. Chromosome Res.

[13] Zanders S, Alani E (2009). The *pch2Δ* mutation in baker's yeast alters meiotic crossover levels and confers a defect in crossover interference.. PLoS Genet.

[14] Joshi N, Barot A, Jamison C, Börner GV (2009). Pch2 links chromosome axis remodeling at future crossover sites and crossover distribution during yeast meiosis.. PLoS Genet.

[15] Wu HY, Burgess SM (2006). Two distinct surveillance mechanisms monitor meiotic chromosome metabolism in budding yeast.. Curr Biol.

[16] Bhalla N, Dernburg AF (2005). A conserved checkpoint monitors meiotic chromosome synapsis in *Caenorhabditis elegans*.. Science.

[17] Li X, Schimenti JC (2007). Mouse pachytene checkpoint 2 (*Trip13*) is required for completing meiotic recombination but not synapsis.. PLoS Genet.

[18] Joyce EF, McKim KS (2009). Drosophila PCH2 is required for a pachytene checkpoint that monitors double-strand-break-independent events leading to meiotic crossover formation.. Genetics.

[19] Borner GV, Barot A, Kleckner N (2008). Yeast Pch2 promotes domainal axis organization, timely recombination progression, and arrest of defective recombinosomes during meiosis.. Proc Natl Acad Sci U S A.

[20] Diaz RL, Alcid AD, Berger JM, Keeney S (2002). Identification of residues in yeast Spo11p critical for meiotic DNA double-strand break formation.. Mol Cell Biol.

